# Chatbot-Based Assessment of Employees’ Mental Health: Design Process and Pilot Implementation

**DOI:** 10.2196/21678

**Published:** 2021-04-21

**Authors:** Ines Hungerbuehler, Kate Daley, Kate Cavanagh, Heloísa Garcia Claro, Michael Kapps

**Affiliations:** 1 Vitalk TNH Health São Paulo Brazil; 2 School of Psychology University of Sussex Brighton United Kingdom; 3 School of Nursing University of Campinas Campinas Brazil; 4 Department of Preventive Medicine University of São Paulo São Paulo Brazil

**Keywords:** chatbot, conversational agent, online, digital health, mobile phone, mental health, workplace, work stress, survey, response rate

## Abstract

**Background:**

Stress, burnout, and mental health problems such as depression and anxiety are common, and can significantly impact workplaces through absenteeism and reduced productivity. To address this issue, organizations must first understand the extent of the difficulties by mapping the mental health of their workforce. Online surveys are a cost-effective and scalable approach to achieve this but typically have low response rates, in part due to a lack of interactivity. Chatbots offer one potential solution, enhancing engagement through simulated natural human conversation and use of interactive features.

**Objective:**

The aim of this study was to explore if a text-based chatbot is a feasible approach to engage and motivate employees to complete a workplace mental health assessment. This paper describes the design process and results of a pilot implementation.

**Methods:**

A fully automated chatbot (“Viki”) was developed to evaluate employee risks of suffering from depression, anxiety, stress, insomnia, burnout, and work-related stress. Viki uses a conversation style and gamification features to enhance engagement. A cross-sectional analysis was performed to gain first insights of a pilot implementation within a small to medium–sized enterprise (120 employees).

**Results:**

The response rate was 64.2% (77/120). In total, 98 employees started the assessment, 77 of whom (79%) completed it. The majority of participants scored in the mild range for anxiety (20/40, 50%) and depression (16/28, 57%), in the moderate range for stress (10/22, 46%), and at the subthreshold level for insomnia (14/20, 70%) as defined by their questionnaire scores.

**Conclusions:**

A chatbot-based workplace mental health assessment seems to be a highly engaging and effective way to collect anonymized mental health data among employees with response rates comparable to those of face-to-face interviews.

## Introduction

On average, people typically spend one-third of their lives at work. Thus, the workplace is one of the key environments that can affect quality of life, and emotional and physical well-being. Generally, work is considered to be good for mental health with involuntary joblessness being a well-recognized risk factor for mental health problems, including depression [1]. However, stressful work conditions can also contribute to the development of mental health problems [2]. Causes of common mental health problems such as depression and anxiety are complex and may include traumatic life experiences or inherited traits but can also be a reaction to work-related stress. The Dunedin Study found that high-demand jobs were associated with the onset of depression and anxiety in people with no prior history of diagnosis or treatment for either disorder [3]. Generally, work-related stress is considered to be a consequence of the organization and management, skills and competencies of employees, and the support they receive [2].

Besides serious consequences for an individual’s mental health and associated direct medical costs, stressful working conditions have indirect costs though reduced productivity due to absenteeism and presenteeism. A recent World Health Organization–led study estimated that depression and anxiety disorders cost the global economy US $1 trillion each year in lost productivity [4]. The implementation of cost-effective and feasible interventions could therefore have a significant impact on the individual, organization, and economy [5].

To minimize the impact of workplace risk factors, adequate policies and intervention programs should be implemented. To determine the appropriate interventions, the current mental health of employees and possible sources of work-related stress must first be assessed. There are specific measures that are commonly used to evaluate different mental health conditions and work-related risk factors, such as the Patient Health Questionnaire-9 (PHQ-9) [6]; Depression, Anxiety and Stress Scale-21 (DASS-21) [7]; and Job Satisfaction Survey (JSS) [8]. Traditionally, these questionnaires are self-administered in person or over the telephone with a health professional who facilitates completion, offering encouragement or clarification as needed. Both approaches yield similar results [9]. It is also possible to complete the scales online, using a webpage or smartphone app to display the questions and collect user responses. This method has been shown to be feasible in both clinical [10] and workplace [11] settings, albeit with relatively low response rates (34%) [11]. Symptom scores reported via a smartphone app strongly correlate with those reported through traditional paper-and-pen methods, although symptoms reported via smartphone were on average 3 points higher [12].

Engagement varies widely among digital health programs and smartphone apps [13]. According to a recent review, low engagement can occur when apps are not designed with the user in mind or do not solve the problem the user cares most about [14]. A lack of interactive or engaging features can also increase the risk of survey fatigue where users become tired and do not finish the survey [14]. Guided self-help interventions have been shown to have greater adherence than nonguided interventions [15]. This suggests that human support could improve engagement; however, this limits scalability. Chatbot-driven conversational surveys could offer an alternative by automating this encouragement and interaction. Chatbots have been shown to have significantly greater engagement and higher-quality responses than typical online surveys [16,17], and the relative anonymity offered by such an approach could be of additional value to employees.

Chatbots, or conversational computer programs, simulate human conversation. Features include word-classification processes, natural language processing, and artificial intelligence in addition to simple keyword scans and databases linking common phrases and predefined responses, which help the chatbot tailor the answers to a specific user input. Most chatbots are accessed via websites or mobile apps, or can be integrated into virtual assistants as a conversational component of a system, which can also control external devices or manage basic tasks such as emails or to-do lists. Chatbots tend to be represented by an animated character, in some cases an embodied “human” conversational agent who uses and responds to verbal and nonverbal communication such as hand gestures or body posture.

Many interactions between organizations and customers are already bot-driven, enabling companies to respond to more people at a faster and cheaper rate than if they use human customer service representatives. Besides being a cost-effective and feasible method of communication, chatbots are capable of generating a believable and dynamic dialogue. This has the potential to enhance engagement rates, using the chatbot to successfully guide and motivate users. Compared to typical online surveys, this may result in a higher level of engagement [16] and greater symptom disclosure [17]. However, to our knowledge, no system of this nature currently exists for assessing workplace mental health in Brazil.

This paper describes the chatbot design process and results of a pilot implementation in a workplace setting. The aim of this study was to explore whether a text-based chatbot is a feasible way to engage and motivate employees to complete a workplace mental health assessment so as to provide important insights for the employee and organization.

## Methods

### Design

This study was a pilot implementation of a chatbot-based mental health assessment performed in a real-world workplace setting, based on a cross-sectional analysis.

### Sample

The sample comprised employees of an industrial plant in São Paulo, Brazil, with a total of 120 employees, who participated in the assessment between October and November 2019. These employees work at the recycling plant (n=52); in reverse logistics operations (n=40); or in the office in administration, information technology, or human resources roles (n=28).

### Approval

All participants provided consent as part of the onboarding process. They agreed for their anonymized data to be used for research purposes and for the aggregated data to be shared with the organization. Data are stored securely and are password-protected.

The company’s Board of Directors approved the use of the routinely collected data for analysis and publication after having been briefed about the assessments and privacy requirements.

Research based on aggregate user data with no possibility of individual identification does not require approval of the Research Ethics Committee (CEP/CONEP) in Brazil.

### Chatbot Development and Testing

**Figure 1:**
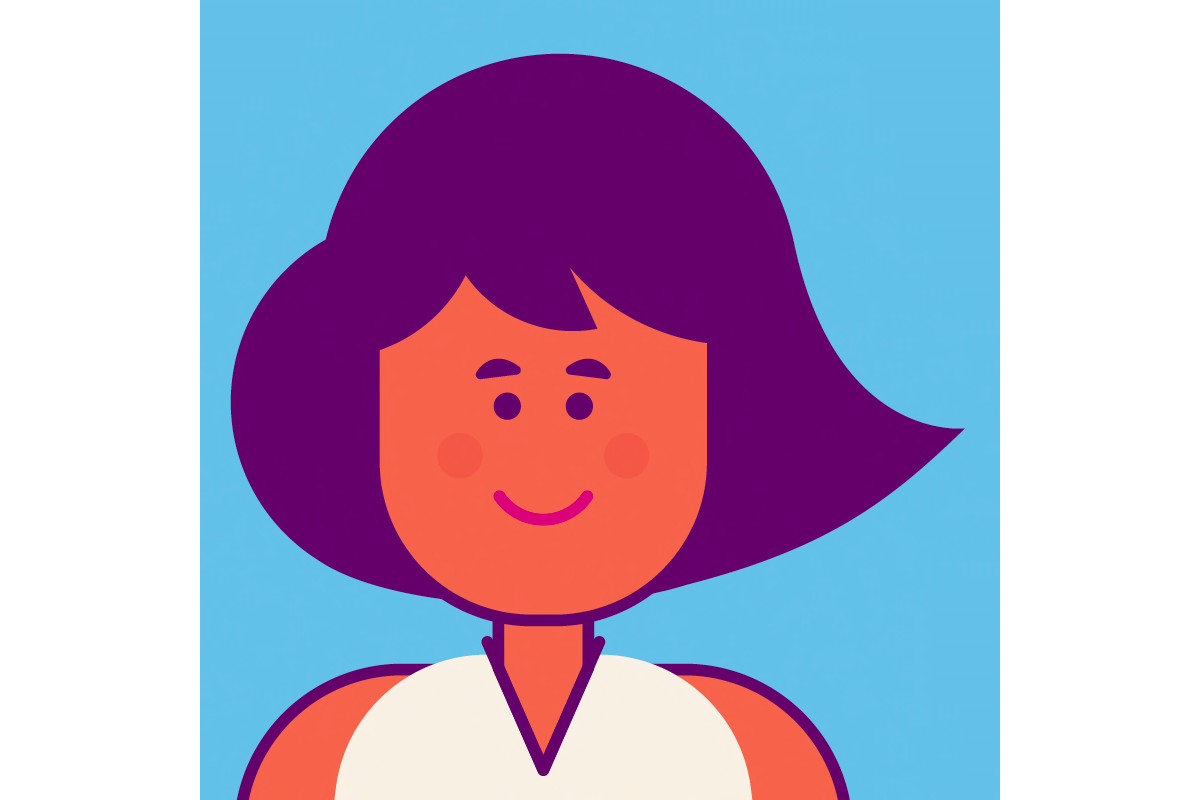
The chatbot avatar Viki.

The chatbot was designed to assess employees’ mental health in a cost-effective and engaging way. This requires a specific developing platform, clinically validated content, and an adequate visual presentation, as well as a clear purpose and a well-defined personality. A key component for reaching this goal is the user experience (UX), which is used to help connect the chatbot with users and build a shared experience. The first step was to analyze the needs, characteristics, and behaviors of the target user group. Based on these insights, the chatbot avatar “Viki” (Figure 1) was created, along with a specific language and conversation style. The UX team performed focus groups and telephone interviews with potential users to verify if the chatbot fits with their expectations and needs. For example, they were asked if the objective was clear, if the length of the checkup was accurate, whether they had any difficulties in completing the checkup, how they rated their experience of communicating with the chatbot, and how they would describe Viki. The data obtained from these interviews were used to refine the chatbot.

The chatbot is built on ruby and javascript, and was created by the team at TNH Health. The checkup assessment is rule-based, with the next steps determined by user responses. As part of the design process, decision trees were defined, and all possible user journeys were mapped and analyzed. Decision trees enabled the chatbot to provide the right responses and information based on the user’s inputs (ie, to customize the conversation). For example, if the user responds that they want to know more about depression, the chatbot delivers further information about the topic. If the user prefers to continue without knowing more about the topic, the chatbot takes them to the next topic. Building a decision tree creation tool with all of the necessary settings for interactions and data organization allowed the chatbot to be updated in an agile way without needing a new system release, which was deemed to be crucial to the development.

**Figure 2:**
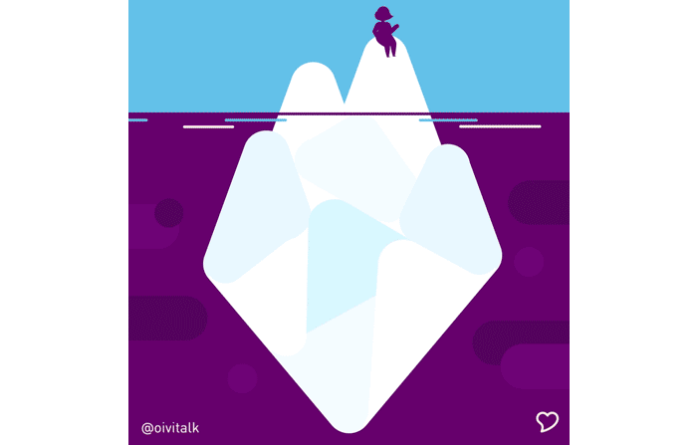
The iceberg story.

Given known challenges in engagement, gamification features were added to address this issue. Gamification is defined as “the use of game design elements in nongame contexts” [18], with features such as levels, challenges, points, progress, feedback, story, and reward [19]. The most important features utilized in this design were story and feedback. For the story, Viki guides users through the assessment process, presented as an expedition around an iceberg (Figure 2). The iceberg represents issues relating to mental health and is divided into different sections: stress, anxiety, depression, burnout, and work-related stress. Insomnia is additionally presented if the user scores positive to the question “I had trouble falling or staying asleep” or “sleeping too much” in the depression block.

**Figure 3:**
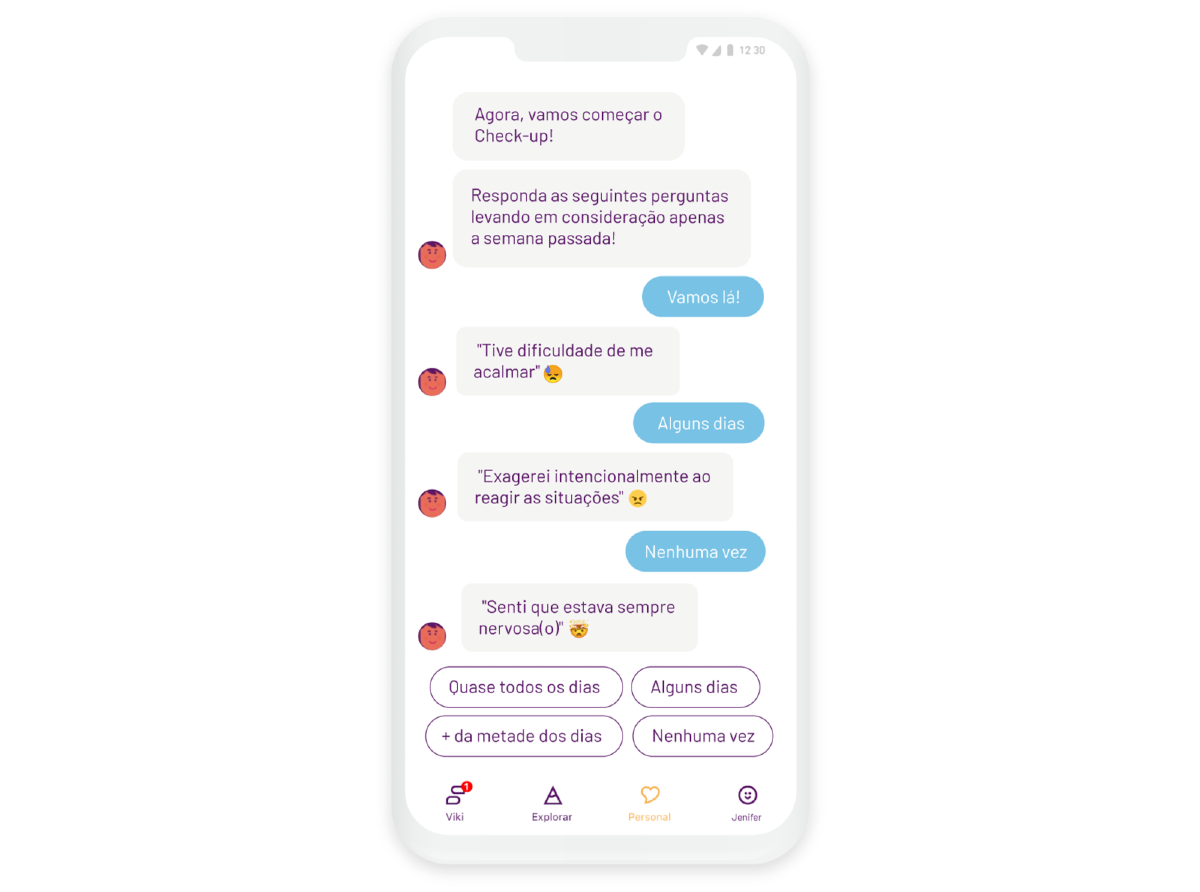
Screenshot of the mental health checkup.

During the conversations, more and more of the iceberg, and thus the key topics, becomes visible to the user. Each section consists of an introduction to the topic and a standardized questionnaire. The questionnaires are delivered in a conversation format, with Viki asking a question and the user selecting their answers from predefined responses using the standard options for each questionnaire (Figure 3). The next question is only presented once the user selects their answer. This is displayed on a rolling screen with approximately three responses visible at a time. The user can go back and change their answer if they want, but there is no functionality included to skip a question. During the checkup, besides the questions for the different scales, Viki also offers messages of encouragement. These messages are designed to keep the participant motivated and engaged, matching the methods real human interviewers use. An example of such a message is “We are almost done, you are doing very well!” All communication is text-based, written in Brazilian Portuguese.

The entire assessment takes approximately 15 minutes. Responses are captured automatically and stored within a secure database. The user issue of nonresponse was discussed in the design process and the team agreed that a limit of 24 hours should be set. The user can pause at any time during the assessment, but if they do not complete the assessment within 24 hours the system will reset and data are overwritten. This ensures that all questionnaire data are collected within a specific time period.

Immediately after completing the assessment, participants receive personalized feedback and recommendations. Participants are reminded that the results do not offer a diagnosis, but may indicate the presence of an emotional problem. In the case of a serious risk identified (ie, very high risk for anxiety or depression or suicide risk detected during the assessment, or via key words typed by users), the conversation follows a safety protocol that includes referral to the care network or, if necessary, to emergency services.

Aggregated and anonymized data are presented on a dashboard and in an organization report to the company’s management. This allows oversight of the assessment process in real time (eg, response rate, distributions of mental health outcome categories of already assessed employees), and offers valuable insights to the organization, identifying issues and recommending actions such as further campaign actions in case of a general low engagement of the employees and planning of target-oriented interventions for specific departments or positions based on the mental health outcome distributions.

Participants are informed within the terms of use, which they are asked to agree to at the beginning of the checkup, about the anonymized sharing of their data with the company. To preserve anonymity, no name or other personal identification data are included on the dashboard or in the report, and departments or sections with less than 8 people were pooled into larger groups.

The usability and technical functionality of the chatbot and dashboards were tested prior to launching with users (with internal staff and organization employees). The only issue raised during this testing was how the chatbot would react to any unexpected free text entered by the employee during the assessment. Therefore, the settings and natural language understanding were adjusted to enable smooth running of the chatbot while adhering to risk protocols.

### Mental Health Outcomes

The checkup includes the following questionnaires to cover the topics of anxiety, depression, stress, burnout, and work-related stress. Insomnia was added for users who did not answer the question about difficulties with sleeping with “Not at all” in the previously applied PHQ-9. All questionnaires were translated and validated in Brazilian Portuguese, with good psychometric properties [20-25]. No changes were made to question order or wording. The Generalized Anxiety Disorder-7 scale is a 7-item self-report scale used to assess anxiety symptoms over the past 2 weeks (eg, “How often have you been bothered by feeling afraid something awful might happen?”). Responses range from 0 (not at all) to 3 (nearly every day). Total scores are divided into four categories: none (0-4), mild (5-9), moderate (10-14), and severe (15+) symptoms [26]. The PHQ-9 is a 9-item self-report scale that evaluates symptoms of depression over the past 2 weeks (eg, “How often have you been bothered by feeling down, depressed, or hopeless?”). Responses range from 0 (not at all) to 3 (nearly every day). Total scores are divided into five categories: none (0-4), mild (5-9), moderate (10-14), moderately severe (15-19), and severe (20+) symptoms [6].

The DASS-21 is a 21-item self-report questionnaire consisting of three scales to measure depression, anxiety, and stress [27]. The stress subscale consists of 7 items and the user is asked how much each statement applied to them in the past week (eg, “I found it difficult to relax”). Responses range from 0 (did not apply) to 3 (applied very much or most of the time). Total scores are doubled and divided into five categories: normal (0-14), mild (15-18), moderate (19-25), severe (26-33), and extremely severe (34+) levels of stress [7].

The Insomnia Severity Index (ISI) is a 7-item self-report questionnaire assessing the nature, severity, and impact of insomnia. Responses range from 0 (no problem) to 4 (very severe problem). Total scores are categorized as: absence (0-7), subthreshold (8-14), moderate (15-21), and severe (22-28) insomnia [28].

The Oldenburg Burnout Inventory is a 16-item self-report scale with two dimensions: exhaustion and disengagement from work (eg, “There are days when I feel tired before I arrive at work”). Answers range from 1 (strongly agree) to 4 (strongly disagree) [29]. For analysis, the following categories were used: very low (0-15), low (16-30), high (31-45), and very high (46+) risk of burnout.

The JSS questionnaire is based on a two-dimensional theoretical model created by Robert Karasek that relates two aspects at work, demands and control, to the risk of illness. A third dimension, support, was later added by Theorell [8] as a protective factor to guard against work-related stress. A 15-item short form of the questionnaire was used to assess work-related risk and protective factors in this pilot (eg, “Does your work demand too much effort? Do you have a choice in deciding how you do your work?”). Scores range from 1 (almost never/totally agree) to 4 (frequently/totally disagree). Categories used for analysis were normal (0-15), slightly increased (16-30), increased (31-45), and extremely increased (46+).

### Recruitment and Implementation

As the first stage of implementation, the mental health assessment was presented to the managers of each department of the company. Subsequently, an internal multichannel information campaign was used to present the assessment to employees and educate them about the importance of mental health. Viki was introduced as part of this campaign, using a variety of online and offline channels (email, intranet, banners, and leaflets). This psychoeducative action aimed to reduce stigma and motivate employees to consider looking at their own mental health, as well as to generate trust in the product.

All employees were then emailed a weblink with an invitation to complete the checkup to obtain feedback on their scores. No financial incentive was offered, and employees were informed that the checkup was anonymous and voluntary. They were told that it would take up to 15 minutes, and no stipulations were made regarding whether to complete the checkup at work or at home. It could be completed via cell phone or computer.

When employees click the link, they first pass through an authentication process where they register and enter their company code (which helped to identify the participants of this specific company survey within the whole user population) and agree to the terms and conditions. Viki then begins the checkup.

### Statistical Analysis

Descriptive statistics are used to summarize sample characteristics, baseline symptoms, and completion rates. Only complete datasets were included in this analysis, and duplicates were not possible due to the registration process used. The Checklist for Reporting Results of Internet E-surveys was used for data reporting [30].

## Results

### Sample and Engagement

Of the 120 eligible employees, 98 employees started the assessment (81.7% initiation rate) and 77 completed it (79% completion rate). This response rate enabled data to be obtained for 64.2% (77/120) of the organization’s workforce. Data collection was performed within 4 weeks. More than half of those responding did so within the first week of the campaign.

Of the 77 employees completing the checkup, the majority were men (45/77, 58%), white (40/77, 52%), and aged between 25 and 44 years (55/77, 71%), reflecting the demographics of the organization’s workforce. The majority of respondents were in occupations of plant and machine operators and assemblers (n=21, 27%) or technicians and associate professionals (n=19, 25%).

### Implementation

During implementation, we noticed differences in the speed of rollout, and considered if the motivation and conviction at a management level could be a factor in engagement. As a preventative measure to maximize engagement, we contacted department managers to emphasize the benefits of the checkup and to troubleshoot any potential issues. No issues were identified, but some managers reported they would ensure that they pass on this information to their teams. The informal feedback received from the lead of the project was that the campaign had been well received and they felt that the chatbot added value. This evidence is of course more anecdotal as it did not form part of the data analysis.

### Mental Health Risks

Overall, the sample scored in the low ranges on the majority of the questionnaires (see Table 1). Scores were higher in the area of work-related stress, with equal control and demand reported alongside lower support. On the PHQ-9, 44% (34/77) of users reported difficulties with sleep and therefore also completed the ISI for insomnia.

For those with symptoms, the majority of respondents scored in the mild range for anxiety (20/40, 50%), mild range for depression (16/28, 57%), subthreshold level for insomnia (14/20, 70%), and moderate level for stress (10/22, 46%) as defined by the questionnaire scores. For burnout, most of the respondents scored in the low-risk category (50/74, 68%), whereas the majority scored in the increased-risk category for job-related stress (53/77, 69%).

**Table 1:** Scores and outcomes for each questionnaire measure.

Measure			Respondents (n)		Mean (SD)		Assessment category
Anxiety (GAD-7^a^)			77		6.21 (4.56)		Low
Depression (PHQ-9^b^)			77		4.40 (5.21)		None
Stress (DASS-21^c^)			77		11.09 (7.13)		Normal
Insomnia (ISI^d^)^e^			34		9.26 (5.66)		Subthreshold
Burnout (OLBI^f^)			77		27.68 (8.38)		Low
**Occupational stress (JSS^g^)**			77		32.38 (3.55)		Increased
	Control	77		12.32 (1.99)		Increased	
	Demand	77		12.19 (1.72)		Increased	
	Support	77		7.86 (2.40)		Increased	

^a^GAD-7: Generalized Anxiety Disorder-7.

^b^PHQ-9: Patient Health Questionnaire-9.

^c^DASS-21: Depression, Anxiety and Stress Scale-21.

^d^ISI: Insomnia Severity Index.

^e^Completed only if sleep was identified as an issue.

^f^OLBI: Oldenburg Burnout Inventory.

^g^JSS: Job Stress Scale.

## Discussion

### Principal Findings

The chatbot-based assessment was successfully implemented in the workplace, suggesting that a chatbot could be a feasible way to engage employees in completing a workplace mental health assessment. There was a 79% (77/98) completion rate, obtaining questionnaire responses from 64.2% (77/120) of the workforce. This compares favorably to face-to-face data collection methods. For example, the São Paulo Megacity study reported a response rate of 81% [31] and epidemiological studies reported a response rate of 70% using the same method of data collection [32]. The completion rate in this analysis is 25% higher than that of results found in a telecommunication company using a web-based screening for depression [11], and is also clearly higher than that of other online surveys where rates below 10% are common [33]. A previous study reported a similar completion rate (78%) using a smartphone app to administer up to three survey sessions per day in a sample of psychiatric outpatients. However, this involved a smaller sample and the participants were financially compensated, which may influence motivation [12]. It would be interesting to further explore the impact of the different components of implementation to ascertain the factors that are most integral for success, such as the chatbot or the onboarding process. An online tool and distribution method could be particularly pertinent in an era of remote working.

The majority of users obtained low scores on all of the questionnaires. However, many users did report symptoms at a moderate or severe level. The proportions scoring at this level for depression (20/77, 26%) and anxiety (12/77, 16%) are higher than the estimated prevalence rates of anxiety disorders (9.3%) and depressive disorders (5.8%) found in the general population of Brazil [34], although these rates do vary considerably depending on the method of data collection and the measure used [35]. Prevalence rates are typically higher in women than in men [35], which is interesting given that this sample predominately comprised men. The risk of burnout identified is similar to levels found in other professional groups such as health workers [36] and teachers [37]. High demand was often reported in combination with a low degree of perceived control, which are both risk factors for burnout. This combination is considered to be the most critical in terms of a negative impact on individual mental health. The presence of anxiety, depression, and insomnia could also impact organizational productivity through absenteeism and presenteeism [2,11], which requires further exploration.

The fact that some people reported symptoms shows the importance of addressing mental health issues within the workplace. It would be interesting to determine whether these people have ever sought professional help or support for these difficulties. Insights gained from the assessment could be used to identify individual- and organizational-level strategies that could be implemented to improve mental health, and potentially productivity, within the workplace.

### Limitations

The questionnaire measures have not yet been validated for use in a chatbot format with gamification. As this pilot showed that implementation is feasible, the next step is to complete a validation study to assess the effect of using this method on the psychometric properties of the questionnaires. Establishing validity is required before conclusions can reliably be drawn regarding the mental health of the workplace.

Although the chatbot could successfully obtain data for 64% of the workforce, there are no data for those who did not participate or for those who dropped out during the assessment. It would be interesting to compare sociodemographic characteristics and baseline symptoms between the groups. For example, previous research indicates that being male, with a lower educational level, and comorbidity of depression with anxiety can increase the risk of dropout and nonengagement [38]. Such insights could be used to adapt the chatbot or onboarding process to make it more appealing to the less-engaged group. Taking age and gender into account has been found to enhance use of digital mental health programs [39].

As this was a cross-sectional data analysis, we do not know if the high response rate will be sustained over time. There is the possibility that the response rate was inflated due to the novelty of the approach, which has been suggested to be a general characteristic of mobile health interventions [40]. Longitudinal research could explore this issue, which could assist in the development of UX with chatbots over time. Additionally, as the system used for output generation (responses to the user’s input) is fixed (based on predefined decision trees), the conversation opportunities are currently limited. A larger amount of conversational data gained over time will be necessary to train the chatbot and make it intelligent in a more autonomous way. This would also allow for free text interpretation, for example.

Without controls, it is unclear if the results are due to the specific sample, their workplace, or the method of data collection, which may facilitate higher levels of disclosure [11]. After validating the questionnaires for use in this format, it would be important to repeat this exercise with different workplaces to ascertain if the results are generalizable to other populations, particularly considering issues of gender, age, work type, and level of education. It would be important to replicate the implementation in a workplace with more gender balance and a larger sample size. A usability questionnaire would further strengthen the validity of the results.

Further research is planned, which will include comparison with traditional paper-and-pen methods and web-based forms. These studies will also include repeat measurement following the implementation of remedial measures to ascertain their impact. We would also like to explore, using implementation science, the factors and processes involved in successful implementation of the chatbot within the workplace since there are many variables involved.

### Conclusions

The creation of healthy workplaces and adequate mental health policies must be based on a comprehensive needs assessment. Face-to-face assessments are not anonymous or scalable, and online surveys are often limited by low response rates. A chatbot offers a fully automated digital solution, incorporating gamification features to engage and motivate employees to complete a workplace mental health assessment. The chatbot was found to have response rates comparable to those of face-to-face interviews, suggesting that this could be a feasible way to collect such data. To further verify this new solution, a validation study comparing it with other formats such as face-to-face interviews or online surveys, as well as including a feasibility and satisfaction analysis, would be the next logical step.

## Abbreviations

DASS-21: Depression, Anxiety and Stress Scale-21
ISI: Insomnia Severity Index
JSS: Job Stress Scale
PHQ-9: Patient Health Questionnaire-9
UX: user experience
